# Olfactory Perception of Oviposition-Deterring Fatty Acids and Their Methyl Esters by the Asian Corn Borer, *Ostrinia furnacalis*

**DOI:** 10.1673/031.009.6701

**Published:** 2009-12-04

**Authors:** Lei Guo, Guo Qing Li

**Affiliations:** Key Laboratory of Monitoring and Management of Plant Diseases and Pests Ministry of Agriculture, Nanjing Agricultural University, Nanjing, China

**Keywords:** antennae amputation, oviposition periodicity, electroantennogram

## Abstract

Olfactory perception of myristic, palmitic, stearic and oleic acids and their corresponding methyl esters by Asian corn borer moths, *Ostrinia furnacalis* (Guenée) (Lepidoptera: Crambidae) was investigated. It was found that mated females with both antennae amputated, in contrast to intact females and females with one antenna removed, could not discriminate between simultaneously provided control filter papers and filters treated with a blend of oviposition-deterring fatty acids. Oviposition by mated females exhibited a very marked periodicity, with all egg masses deposited during the scotophase and most egg masses laid before midnight. According to the peak and trough period of oviposition, electroantennogram (EAG) responses from both mated females and males to the four fatty acids and four methyl esters were tested within two two-hour periods from 3 to 5 hours after the start of darkness and from 1 to 3 hours after light onset, respectively. Significant EAG responses above solvent and background were elicited by all test chemicals from females, and by most of the test compounds from males. EAG values of all test chemicals from mated females were not statistically different between the two test periods except for methyl myristate. Conversely, EAG responses from mated males to myristic acid, stearic acid and their methyl esters significantly differed between the two test periods.

## Introduction

The Asian corn borer, *Ostrinia furnacalis* Guenée (Lepidoptera: Crambidae), is one of the most important pests of corn and cotton in China. *O. furnacalis* neonates tend to feed initially within the whorl of the corn plant, especially on the tassel. When the tassel emerges from the whorl, larvae disperse downward where they burrow into the stalk and the ear, causing a 20% to 80% yield reduction each year in some areas in China (Zhou et al. 1995). *O. furnacalis* larvae may alternatively damage cotton. When they are in cotton plant, the larvae attack terminals and new leaves, flower buds, young bolls and white flowers, or bore into the main stem, fruiting branches and green bolls, resulting in a severe loss of squares, green bolls and yield (Zhou et al. 1995; He et al. 2006).

Control of *O. furnacalis* using insecticides is difficult as the larvae are vulnerable to sprays for only a short time before they tunnel into the stalks, squares, or bolls. This makes the timing of insecticide applications crucial for successful control (Zhou et al. 1995; He et al. 2006). Moreover, reduction in insecticide use is becoming increasingly important for economic, ecological and environmental reasons. Therefore, a new system of integrated pest management strategy must be developed in order to achieve an acceptable and cost effective method of *O. furnacalis* control.

Ethological control methods such as application of infochemicals to disrupt feeding, mating and oviposition behaviors may have an essential role in such a system (Shelton and Badenes-Perez 2006; Cook et al. 2007; Zehnder et al. 2007). This entails careful study of olfactory behaviors, as well as the identification and evaluation of these infochemicals involved in insect communication. Our previous results showed that larval frass of *Ostrinia scapulalis, O. furnacalis, O. latipennis* and *O. zealis* (Li and Ishikawa 2004) or egg masses of *O. scapulalis* and *O. furnacalis* (Li and Ishikawa 2005) sandwiched between the layers of a piece of cotton significantly restrained female moths from ovipositing. The main chemical components of oviposition deterrents in the larval frass or on the egg masses of *Ostrinia* species are myristic, palmitic, palmitoleic, stearic, oleic, linoleic and linolenic acids (Li and Ishikawa 2004, 2005). These results indicate that the oviposition-deterring fatty acids in the larval frass or on the surface of eggs are volatile and are probably detected by female antennae. The current study was undertaken to ascertain (1) whether these fatty acids were detected by moth antennae, (2) whether these fatty acids and their methyl esters could elicit strong EAG responses from both females and males, and (3) whether there were any diurnal variations in sensitivities of EAG responses to these compounds.

## Materials and Methods

### Insects

*Ostrinia furnacalis* egg masses were collected in maize, *Zeamays* L. (Poales: Poaceae) field at Nanjing (32.0N, 118.5E), Jiangsu Province in China in July and August 2006, and routinely reared in an insectary under controlled temperature (28±1°C), photoperiod (14 L:D) and relative humidity (70–80%) until pupation. Newly emerged moths (15 females and 15 males) were caged in an open 4500 cm plastic container, given unlimited access to a 10% sucrose solution and were allowed to mate for two consecutive nights. The container was then covered with wax paper, which the moths used as an oviposition substrate. Almost all egg masses were laid on the wax paper during the scotophase. The paper with egg masses was changed every other day, sterilized according to the method described previously by Xu et al. (2006) and egg masses were transferred individually into a plastic box (15 cm in diameter and 6 cm in height). Between 12 and 24 h prior to egg hatching, an artificial diet was offered. The diet contained wheat-germ powder (27.5 g), maize flour (7.5 g), yeast (2.0 g), agar (2.5 g), water (93 ml), minerals, vitamins and citric acid. The larvae were kept in the plastic box until pupation. Females and males were then segregated based on the morphology of the terminal abdominal segments of the pupa.

To obtain experimental moths, sexed pupae were removed to an insectary with a reversed light cycle (14 h light/10 h dark, light off from 09:00AM to 19:00 PM). After emergence, moths (20 females and 20 males) were caged in an open 4500 cm^3^ plastic container, given unlimited access to a 10% sucrose solution, and allowed to copulate. Mating couples were removed from the cage carefully and transferred individually into a 100 ml glass jar. These mated moths were used for bioassays and EAG tests.

### Chemicals

Tetradecanoic acid (myristic acid, C_14:0_), hexadecanoic acid (palmitic acid, C_16;0_), (Z)-9-hexadecenoic acid (palmitoleic acid, C_16:1_), octadecanoic acid (stearic acid, C_18:1_), (Z)-9-octadecenoic acid (oleic acid, C_18:1_), (Z,Z)-9,12-octadecadienoic acid (linoleic acid, C_18:2_), (Z,Z,Z)-9,12,15-octadecatrienoic acid (linolenic acid, C_18:3_) and tridecyl acetate were purchased from Sigma (www.sigmaaldrich.com), and all had claimed purities of 99%. Methyl myristate (C_14:0_ME), methyl palmitate (C_16;0_ME), methyl stearate (C_18:0_ME) and methyl oleate (C_18.1_ME) were synthesized via the methylation of corresponding fatty acids as follows. Ten µg of individual fatty acid was mixed with 5 ml of 5% H_2_SO_4_ in methanol, and the mixture was kept at 90°C for 2°h. Then, 10 ml of hexane was added to the mixture and shaken vigorously. The hexane layer was obtained, dried under a stream of nitrogen and quantified by GC analysis with tridecyl acetate as an internal standard according to the method of Li and Ishikawa (2004, 2005). Chemicals were kept refrigerated between experimental sessions.

### Preparation of Test Materials: The Blend Ratio

Authentic fatty acids were dissolved in analytical grade hexane to prepare following solutions: (1) a blend of C_16:0_ (7.5 µg/ml), C_16:1_ (7.5 µg/ml), C_18:0_ (2.5 µg/ml), C_18:1_ (6.5 µg/ml), C_18:2_ (4.5 µg/ml) and C_18:3_ (1.5 µg/ml) in the ratio found in the extract of *O. furnacalis* egg masses (Blend Ratio) (Li and Ishikawa 2005), (2) 5 µg/µl solution of C_14:0_, C_16:0_, C_18:0_, C_18:1_, C_14:0_ME, C_16:0_ME, C_18:0_ME or C_18:1_ME.

### Dual-choice bioassay

The dual-choice bioassay took place under the environmental conditions outlined for larval rearing. In order to test perception of the Blend Ratio by mated females, three groups of mated females were prepared: intact females, females with one antenna removed after copulation, and females with two antennae removed after copulation. For antennal removal, females (3 days old) were anaesthetized with carbon dioxide for an average 20 seconds until they did not move anymore. The antenna was then cut from the base with micro-scissors. In order to prevent a biased result due to possible effects of anaesthetization, moths of the three groups were anaesthetized and handled in the same way as in antennal removal treatments, except for the actual amputation of the antennae.

The dual-choice bioassay was performed according to the method of Li and Ishikawa (2005). Briefly, 0.2 ml solution of the Blend Ratio and the same amount of hexane were, respectively, applied to two pieces of filter papers (10 × 10 mm) as treatment and control. Two hours before the start of scotophase, a treatment and three controls were placed on top of the fabric screen cage (20 × 20 × 20 cm), each near the midpoint of the four sides, and then overlaid with four square plastic plates (20 × 20 × 1 mm). A mated female was allowed to oviposit for one night, and the numbers of the egg masses and the eggs in each egg mass laid on a treatment plate (T) and three control plates (C) were counted the following morning. The effect of removal of antennae on oviposition was evaluated using the total number of egg masses, and the mean eggs per egg mass, laid by each female on both treatment and control plates. The oviposition behavior in each test was quantified according to an avoidance index (Ai): Ai = (C-3T)/(C+3T) that measured whether the female avoided laying on a plate. The difference between treatments and control was examined using Student's *t*-test at α = 0.05.

### Diurnal pattern of oviposition

A no-choice bioassay was carried out under the environmental conditions outlined for larval rearing. Ten mated females were introduced into an open 4500 cm^3^ plastic container covered with wax paper as an oviposition substrate, given unlimited access to a 10% sucrose solution and allowed them to oviposit for four consecutive photoperiods. The number of the egg masses deposited on wax paper was counted at 2-hour intervals under a red 10-watt light bulb that was located 2 m from the cages (unpublished data of the authors indicate that mated females do not respond to red light). The number of the egg masses served as evidence of the number of times oviposition had taken place during each 2-hour interval.

### Electroantennogram (EAG) Analyses

EAG responses from isolated antennae were performed according to the methods described previously by Xu et al. (2006), with slight modification. Electroantennogram measurements were made using a commercially available electroantennographic system (Syntech, www.syntech.nl). Mated females and males (3–4 days old) were anaesthetised with carbon dioxide for about 20 seconds and the antennae were excised from the head with micro-scissors within two two-hour periods from 3 to 5 hours after the start of darkness under a red 10-watt light bulb, and from 1 to 3 hours after light onset, in accordance with the peak and trough period of oviposition respectively. An isolated antenna was connected between two stainless steel electrodes on the MTP-4 probe using Spectra 360 conductive gel (Parker Laboratories, Inc., www.parkerlabs.com). The EAG signals were amplified and monitored with a head-stage preamplifier and further amplified and processed with a PC-based signal processing system (Syntech).

Odors were generated from a number of chemicals. These chemicals were dissolved in analytical grade hexane respectively to prepare a 5 µg/µl solution. For each compound, an aliquot (10 µl) of solution was applied onto a piece (5 × 30 mm) of filter paper strip. The filter paper was then placed in a glass Pasteur pipette (15 cm long). The tip of the pipette was inserted into a small hole (3 mm diameter, 12 cm from the outlet) of a main airflow tube (12 mm diameter, 17 cm long) in which a continuous, charcoal filtered and moistened airflow (4 ml/s) was blown through onto the prepared antenna. A 0.5-s puff of charcoal-filtered airflow (4 ml/s) was injected through the large end of the Pasteur pipette, transporting the volatiles from the test chemical to antenna for stimulation, using an electronically controlled stimulus flow controller (CS05, Syntech).

Each compound was tested three times as following protocol: C_14:0_ME, hexane blank, compound, hexane blank, C_14:0_ME at 1 min intervals between tests to allow disadaptation of the prepared antenna. Each series of test compounds was repeated in a randomized order with ten different EAG preparations. The mean EAG response elicited by each chemical was compared with that by solvent control and background with a paired *t* test.

To control for variation in responses among antennae and to compensate the decline in antennal sensitivity during a measuring session, the absolute EAG responses to 50 µg C_14:0_ME on filter paper strip were scaled to a value of 1 and 0.58 from mated male in photo- and scotophase and to 0.06 and 0.09 from mated female in photo- and scotphase according to the ratio of mean absolute EAG values. The values obtained between two 50 µg C_14:0_ME calibration references were calculated by linear interpolation. To account for solvent and other background effects when compared the difference between the two test periods and among the test chemicals, we subtracted the averaged EAG responses to hexane recorded before and after each test chemical as described by Dickens (1984). Thus, the corrected EAG response = Rc-[(R_c-1_+R_c+1_)/2], where R_c_ is a single EAG response elicited by a test chemical, R_c-1_ is the response to hexane before the test chemical and R_c+1_ is the response to hexane after the test chemical. The difference between the two test periods in the mean normalized responses of each of the test chemicals was compared by Student's *t* test and the differences of the mean normalized responses among the test chemicals in each of the two test periods were submitted to ANOVA and compared by Duncan's multiple range test (Duncan 1955).

**Table 1.**
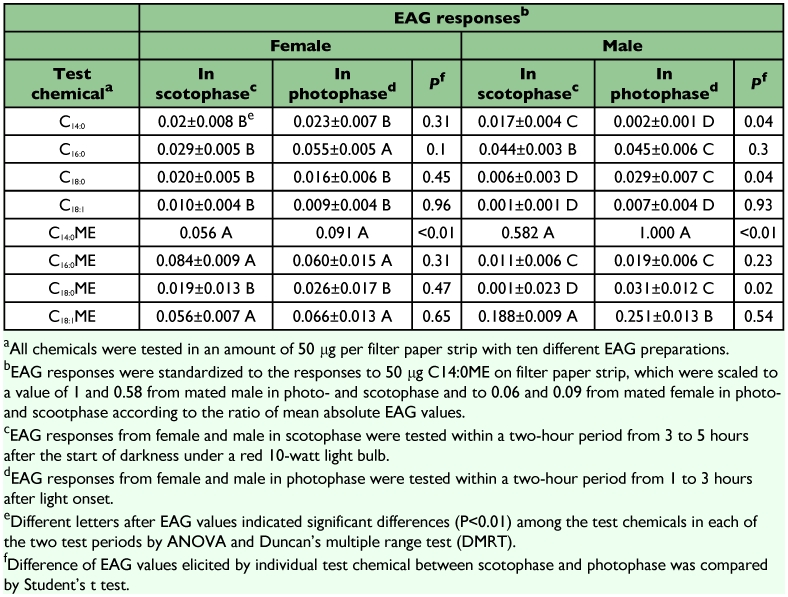
EAG Responses elicited by the test chemicals from Ostrinia furnacalis mated moths.

## Results

### Effect of antennae removal on oviposition deterrent detection

**Figure 1.**
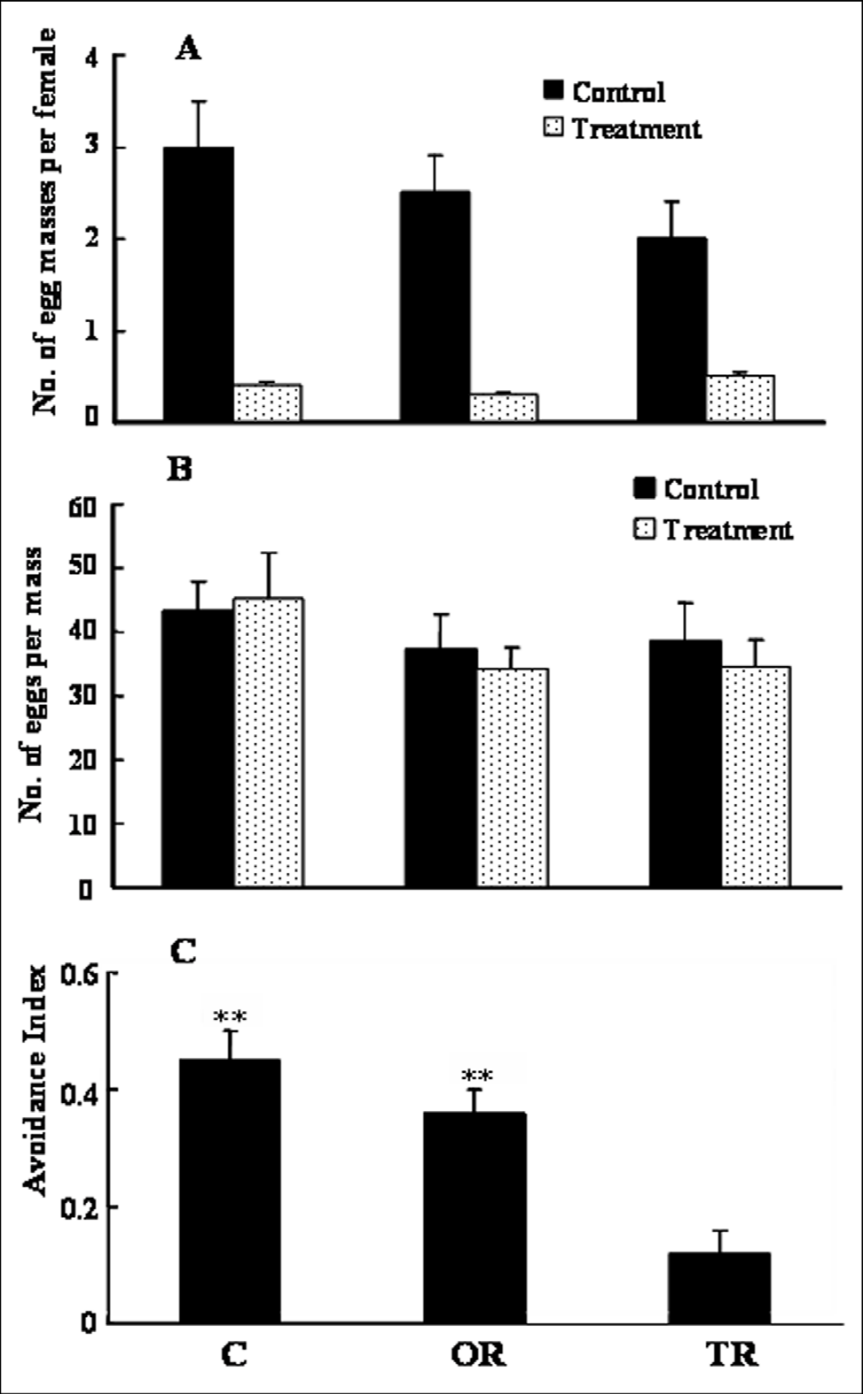
The effect of antennectomization on oviposition by *Ostrinia furnacalis*. The total egg masses laid on one treatment and three controls by each mated female with intact antennae (C), one antenna removed (OR), and two antennae removed (TR) were 4.2, 3.6 and 3.4, respectively. 1A) Effects of removal of antennae on the number of laid egg masses. 1 B) The mean eggs per egg mass laid on both treatment and control by each C, OR, or TR mated females. 1 C) The ability of mated females to discriminate between simultaneously provided control filter papers and filters treated with the Blend Ratio after the ablation of one or two antenna(e). (C) intact females, (OR) females with one antenna removed (TR) females with both antennae removed.

The effect of removal of antenna on oviposition was tested. The average total egg masses laid on one treatment and three controls by each mated female with intact antennae, one antenna removed, and two antennae removed was 4.2, 3.6 and 3.4, respectively. Only small effects of removal of antennae on the number of laid egg masses were observed (Figure 1 A). Similarly, the mean number of eggs per egg mass laid on both treatment and control plates by control or mated females after removal of antennae showed little difference (Figure 1 B).

The ability of mated females to discriminate between simultaneously provided control filter papers and filters treated with the Blend Ratio was studied after the ablation of one or two antenna(e). Intact females and females with one antenna removed preferred to lay their eggs on control filters. Females with both antennae removed, however, deposited similar numbers of egg masses on control and treated filters (Figure 1 C).

### Diurnal oviposition pattern

**Figure 2.**
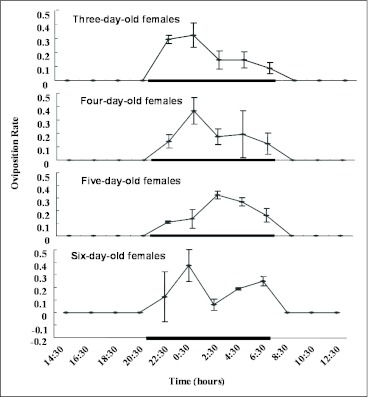
Daily oviposition patterns of *Ostrinia furnacalis* in a no-choice bioassay. Ten mated females were introduced into an open 4500 cm3 plastic container covered with wax paper as oviposition substrates and allowed to oviposit for four consecutive photoperiods with a scotophase of 10 hours from 21:00 P.M. to 7:00 A.M. as indicated. The egg masses deposited on wax paper were counted at 2-hour intervals. The oviposition rate was the number of egg masses deposited within each 2-hour interval divided by the total number of egg masses oviposited in each light cycle. Oviposition rates represent the mean ± SE and average from 4 replicates.

The number of egg masses laid during each two-hour period throughout 4 consecutive photoperiods was recorded (Figure 2). Oviposition by *O. furnacalis* mated females exhibited a very marked periodicity. All egg masses were laid during scotophase and most egg masses were deposited before midnight.

### EAG responses of females evoked by authentic fatty acids and corresponding methyl esters

Olfactory perception of C_14:0_, C_16:0_, C_18:1_, C_18:1_ and their corresponding methyl esters by mated females were evaluated by EAG responses within a two-hour period from 3 to 5 hours after the start of darkness, in accordance with the peak period of oviposition. Each of the test chemicals elicited significant EAG response above solvent and background (C_14:0_*P*=0.0013, C_16:0_*P*=0.0387, C_18:0_*P*=0.0195, C_18:1_*P*=0.0021, C_14:0_ME *P*<0.0001, C_16:0_ME *P*=0.0041, C_18:0_ME *P*= 0.0044 and C_18:1_ME *P*=0.0028, by a paired *t* test. Data not shown) when applied in an amount of 50 µg per filter paper strip. C_14:0_ME, C_16:0_ME and C_18:1_ME evoked higher EAG responses than C_14:0_, C_16:0_, C_18:0_, C_18:1_, and C_18:0_ME (Table 1).

Similarly, EAG responses from mated females to all test chemicals within a two-hour period from 1 to 3 hours after light onset, in accordance with the trough period of oviposition, were significantly higher than response to hexane blank and background (C_14:0_*P*=0.0067, C_16:0_*P*=0.0232, C_18:0_*P*=0.0055, C_18:1_*P*=0.0075, C_14:0_ME *P*<0.0001, C_16:0_ME *P*=0.0018, C_18:0_ME *P*=0.0337 and C_18:1_ME *P*<0.0001, by a paired *t* test. Data not shown). C_16:0_, C_14:0_ME, C_16:0_ME and C_18:1_ME released stronger EAG responses than C_14:0_, C_18:0_, C_18:1_, and C_18:0_ME (Table 1).

Difference in EAG responses to each of the test chemicals between the two test periods was compared. EAG response to C_14:0_ME was significantly weaker in the test period of scotophase (Table 1).

### EAG responses of males evoked by authentic fatty acids and corresponding methyl esters

The EAG response from mated males within a two-hour period from 3 to 5 hours after the start of darkness to C_14:0_, C_16:0_, C_14:0_ME, C_16:0_ME or C_18:1_ME was significantly higher than the response to hexane blank and background (C_14:0_*P*=0.0044, C_16:0_*P*=0.0009, C_14:0_ME *P*<0.0001, C_16:0_ME *P*=0.0004 and C_18:1_ME *P*=0.0016 by a paired *t* test. Data not shown). Among the test compounds, C_14:0_ME and C_18:1_ME evoked highest and sub-highest EAG responses and C_16:0_ followed. C_18:0_, C_18:1_ and C_18:0_ME released weak EAG responses, similar to hexane blank and background (Table 1).

Similarly, EAG response from mated males to C_16:0_, C_18:0_, C_14:0_ME, C_16:0_ME, C_18:1_ME or C_18:1_ME within a two-hour period from 1 to 3 hours after light onset was stronger than that to hexane blank and background (C_16:0_*P*=0.002, C_18:0_*P*=0.01, C_14:0_ME *P*<0.0001, C_16:0_ME *P*<0.0001, C_18:0_ME *P*=0.0055 and C_18:1_ME *P*=0.002, by a paired *t* test. Data not shown). Among the test compounds, C_14:0_ME released strongest EAG response and C_18:1_ME followed. C_16:0_, C_14:0_ and C_18:1_ released low EAG responses, comparable to solvent and background (Table 1).

Difference in EAG responses from mated males to each of the test chemicals between the two test periods was compared. EAG response to C_14:0_ was significantly stronger in the test period in scotophase. In contrast, EAG responses to C_18:0_, C_14:0_ME and C_18:0_ME were significantly weaker in the test period in scotophase (Table 1).

## Discussion

Our previous results showed that larval frass or egg masses from *Ostrinia* species sandwiched between the layers of a piece of cotton significantly reduced female moths oviposition. The chemical components of the oviposition deterrents originated from both larval frass and egg masses were C_14:0_, C_16:0_, C_16:0_, C_16:1_, C_18:1_, C_18:2_ and C_18:3_ (Li and Ishikawa 2004, 2005). This indicates that the oviposition-deterring fatty acids are volatile and may be detected by female antennae. Consistent with this indication, in the navel orangeworm *Amyelois transitella*, C_18:1_ and C_18:2_ provide the cues used for flight orientation toward the source of an odor in a wind tunnel (Phelan et al. 1991). Moreover, similar oviposition-deterring fatty acids and their blends elicit significant EAG responses and typical dose-response curves in the cotton bollworm, *Helicoverpa armigera* (Xu et al. 2006). In the present paper, we found females with both antennae amputated, in contrast to intact females and females with one antenna removed, could not discriminate between simultaneously provided control filter papers and filters treated with a blend of oviposition-deterring fatty acids. This implies that the oviposition-deterring fatty acids are perceived by *O. furnacalis* antennae. Similarly, several phytophagous pests such as *Spodoptera littoralis* (Hilker and Klein 1989; Anderson et al. 1993), *Pieris brassicae* (Behan and Schoonhoven 1978), *P. rapae* (Schoonhoven 1990), *Ceutorhynchus assimilis* (Ferguson et al. 1999) and *Callosobruchus subinnotatus* (Mbata and Ramaswamy 1995), and parasitoid species such as *Hipster horticola* (van Nouhuys and Ehrnsten 2004), *Pteroptrix longiclava* and *Encarasis gigas* (Chi et al. 1997), *Anaphes iole* (Conti et al. 1997; Wu and Nordlund 2002), *Epidinocarsis lopezi* and *Leptomastix dactylopii* (van Baaren and Nenon 1996), *Trichogramma evanescens* (Salt 1937) and *Trissolcus basalis* (Colazza et al. 1996; Field 1998; Rosi et al. 2001) have been reported to perceive oviposition-deterring pheromones by antennae.

The electroantennogram technique measures the summation of receptor potentials from responding cells in insect antennae. This technique is much easier than behavioral assays or single sensillum recordings and up to now remains a powerful method in infochemical researches (Burguiere et al. 2001; Wibe 2004; Bruce et al. 2005). The EAG technique was used to estimate the detection by both female and male *O. furnacalis* moths of C_14:0_, C_16:0_, C_18:0_ and C_18:1_ and their corresponding methyl esters, that have been shown to be the oviposition-deterring pheromones in the extract of eggs of *Ostrina nubilalis* (Thiéry and Le Quere 1991). The results showed that significant EAG responses above solvent and background were elicited by all test chemicals from females and by most of the test compounds from males. Among the three saturated fatty acids, C_16:0_ gave the strongest EAG response to both mated females and males. The EAG responses of these compounds are not proportional to volatility, demonstrating that the carbon length evidently affects their activities. Similarly, it has been reported that many insects are more responsive to C_6_ straight-chain compounds and to a lesser extent to C_5_-, C_7_- and C_8_ straightchain compounds, irrespective of the terminal functional groups (Visser 1979; Kozlowski and Visser 1981; Dickens 1984; Light et al. 1988; Burguiere et al. 2001). Between two octadecanoyl species, the saturated fatty acid evoked higher EAG responses than unsaturated fatty acid. In contrast, the saturated methyl ester elicited lower EAG response than unsaturated esters. Influences of unsaturated bonds on EAG responses have been also documented by Guerin and Städler (1982), Visser (1983,1986), Light et al. (1988) and Burguiere et al. (2001). Methylation of fatty acids can improve their volatility, the EAG responses to C_16:0_ME and C_18:0_ME, however, were not always increased by methylation. These results demonstrated that the carbon length, double band and methylation obviously affect the activities of the test chemicals.

It was evident that C_14:0_ME and C_18:1_ME triggered the highest and the second highest EAG responses in both scotophase and photophase by males among test fatty acids and their methyl esters. In contrast, EAG responses caused by C_14:0_ME in both scotophase and photophase from females were similar to those elicited by some of the fatty acids and methyl esters tested. It seems that a type of specific sensilla in male antennae is responsible for the detection of C_14:0_ME and C_18:1_ME, the esters with a 14-carbon hydrophobic or unsaturated hydrophobic chain. In this regard, the sex pheromone sensilla in male antennae, were shown to be sensitive to (Z) and (E)-12-tetradecenyl acetates that are also esters with a 14-carbon unsaturated hydrophobic chain (Takanashi et al. 2006). Further investigations, especially wind tunnel experiments and single-sensillum recording, are needed to confirm this.

*Ostrinia* species are nocturnal moths and lay their egg masses at night. Oviposition of *O. nubilalis*, for example, showed a 24-hour rhythm with a majority of egg masses deposited shortly before midnight (Schurr and Holdaway 1966). Our results showed that oviposition by *O. furnacalis* females also exhibited a very marked periodicity. All egg masses were laid during the scotophase and most egg masses were deposited before midnight. It is of substantial interest to ascertain whether there are any diurnal variations in olfactory sensitivities in moths to oviposition-deterring fatty acids and corresponding methyl esters between the peak vs. the trough period of oviposition. Our result revealed that C_14:0_ME exhibited obvious variations in EAG amplitudes from *O. furnacalis* females. Similarly, differences in EAG responses by mated males to C_14:0_, C_18:0_, C_14:0_ME and C_18:0_ME between the two test periods were found. The biological importance of the sexual difference in the antennal sensitivity to these fatty acids and corresponding methyl esters needs further research.

## Abbreviations

EAG: electroantennogram
Blend Ratio: ratio of fatty acids found in the extract of *O. furnacalis* egg masses
